# Identify the Risk Factors of COVID-19-Related Acute Kidney Injury: A Single-Center, Retrospective Cohort Study

**DOI:** 10.3389/fmed.2020.00436

**Published:** 2020-07-28

**Authors:** Jing Wang, Zhixian Wang, Yunpeng Zhu, Haichao Li, Xiaoning Yuan, Xiaoning Wang, Yuxi Wang, Jinqian Hu, Chunxiang Feng, Chang Liu, Shiliang Liu, Kai Yu, Xing Li, Xiaoyong Zeng

**Affiliations:** ^1^Department of Urology, Tongji Hospital, Tongji Medical College, Huazhong University of Science and Technology, Wuhan, China; ^2^Department of Pulmonary, Peking University First Hospital, Beijing, China; ^3^Department of Hospital-Acquired Infection Control, Peking University Third Hospital, Beijing, China; ^4^Department of Thoracic Surgery, Shanxi Bethune Hospital, Taiyuan, China; ^5^Department of Nephropathy, Tongji Hospital, Tongji Medical College, Huazhong University of Science and Technology, Wuhan, China; ^6^Department of Anesthesiology, Tongji Hospital, Tongji Medical College, Huazhong University of Science and Technology, Wuhan, China; ^7^Department of Urology, Union Hospital, Tongji Medical College, Huazhong University of Science and Technology, Wuhan, China; ^8^Department of Geriatrics, Tongji Hospital, Tongji Medical College, Huazhong University of Science and Technology, Wuhan, China; ^9^Department of Ultrasonography, Tongji Hospital, Tongji Medical College, Huazhong University of Science and Technology, Wuhan, China

**Keywords:** acute kidney injury, COVID-19, risk factor, procalcitonin, glomerular filtration rate, retrospective cohort study

## Abstract

**Background:** The kidney is a target organ that could be infected by SARS-CoV-2, and acute kidney injury (AKI) was associated with a higher risk of COVID-19 patients' in-hospital death. However, no published works discussed about the risk factors of COVID-19 related AKI.

**Methods:** We conducted a retrospective cohort study, recruiting COVID-19 inpatients from the Sino-French branch of Tongji Hospital. Demographic, clinical, treatment, and laboratory data were collected and compared. We used univariable and multivariable logistic regression methods to identify the risk factors of COVID-19-related AKI.

**Results:** Of the 116 patients in our study, 12 (10.3%) were recognized as AKI, including 5 (4.3%) in-hospital AKI. Multivariable regression showed increasing odds of COVID-19-related AKI associated with COVID-19 clinical classification (OR = 8.155, 95% CI = 1.848–35.983, ref = non-critical, *p* = 0.06), procalcitonin more than 0.1 ng/mL (OR = 4.822, 95% CI = 1.095–21.228, *p* = 0.037), and estimated glomerular filtration rate (eGFR) <60 mL/min/1.73 m^2^ (OR = 13.451, 95% CI = 1.617–111.891, *p* = 0.016).

**Conclusions:** COVID-19-related AKI was likely to be related to multiorgan failure rather than the kidney tropism of SARS-CoV-2. The potential risk factors of COVID-19 clinical classification, procalcitonin more than 0.1 ng/mL, and eGFR <60 mL/min/1.73 m^2^ could help clinicians to identify patients with kidney injury at an early stage.

## Introduction

At the close of 2019, several patients with pneumonia of unknown cause were detected in Wuhan, China, and first reported to the World Health Organization Country Office in China on Dec 31, 2019 (1). Subsequently, the pathogen was identified as a novel enveloped RNA betacoronavirus (2) and named as “severe acute respiratory syndrome coronavirus 2” (SARS-CoV-2) by the Coronavirus Study Group (CSG) of the International Committee on Taxonomy of Viruses (3). On Feb 11, 2020, WHO officially announced the disease caused by SARS-CoV-2 as “Coronavirus Disease 2019” (COVID-19) (4). Given the alarming levels of spread and severity of COVID-19, WHO characterizes COVID-19 as a pandemic (5), causing a total of 9,619,573 confirmed cases and 489,556 deaths globally as of June 26, 2020 (6).

Angiotensin-converting enzyme 2 (ACE2) was confirmed as the receptor that mediates the entry of SARS-CoV-2 into human cells, and the affinity of ACE2 protein with SARS-CoV-2 was 10–20 times that of SARS-CoV (7–9). The ACE2-expressing level in human tissues, using the RNA-seq method, showed that the level in urinary organs (kidney) was much higher (nearly 100-fold) than that in respiratory organs (lung) (10). In the kidney tissue, glomerular visceral ACE2 staining was observed (11). Theoretically, the kidney is a possible target organ that could be infected by SARS-CoV-2.

In this study, we reported the clinical characteristics of COVID-19 patients admitted to our center, focusing on kidney injury. We aimed to characterize the clinical characteristics and identify the potential risk factors of COVID-19-related AKI, in order to facilitate clinical management of COVID-19.

## Materials and Methods

### Study Design and Participants

We designed a retrospective cohort study. In-hospital patients with COVID-19 were pooled from the Sino-French branch of Tongji Hospital of Tongji Medical College, Huazhong University of Science and Technology between Mar 9, 2020, and Mar 17, 2020. The Sino-French branch of Tongji Hospital, located in Wuhan, Hubei Province, was assigned responsibility for the treatments of severe COVID-19 patients by the Wuhan government on Jan 25, 2020. The hospital's wards were reprogrammed into a number of dedicated COVID-19 treatment units and taken over by medical teams from other parts of the country.

All patients who were enrolled in this study were diagnosed as COVID-19 according to the guidance provided by the Chinese National Health Commission (12). Patients with a history of maintenance dialysis, chronic kidney disease, or renal transplantation were excluded. The clinical observation was monitored up to Mar 17, 2020.

### Data Collection

Data collection was conducted by two independent researchers (XYZ and JW), using a standardized data collection form. We extracted epidemiological, demographic, clinical, laboratory, treatment, and outcome data from electronic medical records. In order to track the changes in renal function of patients during hospitalization, we retrospectively recorded the laboratory findings of renal function in detail in chronological order. The researchers, ZXW and XL, adjudicated any discrepancy in interpretation between the two primary researchers and tracked the missing core data.

### Definition

Acute kidney injury (AKI) was diagnosed and classified according to the Kidney Disease: Improving Global Outcomes (KDIGO) criteria clinical practice guidelines (13). AKI was defined as an increase in serum creatinine (sCr) by 0.3 mg/dL (26.5 μmol/L) within 48 h or a 50% increase in sCr from the baseline within 7 days. The stage of AKI was classified by the sCr level, with increased 1.5–1.9, 2.0–2.9, and ≥3 times than baseline being defined as stages 1, 2, and 3, respectively (13, 14). Serum creatinine elevated was recognized as ≥104 μmol/L for males and ≥84 μmol/L for females, based on the reference range given by the Department of Laboratory of Tongji Hospital. In-hospital AKI was defined as the patient with normal sCr at admission, who developed AKI after admission. The clinical classification of COVID-19 was defined according to the Chinese management guideline for COVID-19 (trial version 7.0) (12). The general case was defined as patients with fever and other mild respiratory symptoms, combining imaging findings of pneumonia. The severe case was defined as either (i) polypnea, respiratory rate >30 per min, or (ii) oxygen saturation ≤93%, or (iii) PaO_2_/FiO_2_ ratio ≤300 mmHg. The critical case was defined as meeting any criterion of the following: respiratory failure requiring mechanical ventilation; shock; and organ failure requiring admission to intensive care unit (ICU). Exposure history was defined as ever exposed to people with certain SARS-CoV-2 infection or the Wuhan Huanan Seafood Market. Coagulation disorder was defined as a 3-s extension of prothrombin time or a 5-s extension of activated partial thromboplastin time (15).

### Statistical Analysis

Continuous and categorical variables were presented as median (interquartile range) and n (%), respectively. To compare differences between AKI group and non-AKI group, we used the Mann–Whitney *U*-test (for continuous data), Pearson's χ^2^-test, or Fisher's exact test (for categorical data) where appropriate. The variables that were statistically significant (*p* < 0.1) in the comparison of the two groups were analyzed by univariable logistic regression firstly, for screening the potential risk factors associated with AKI. The continuous variables (IL-2R, IL-6, TNF-α, PCT, hs-CRP, ESR, platelet count, albumin, NT-proBNP, hs-cTnI, eGFR) were converted into categorical ones, according to the normal reference range and the previous published literature (15, 16). Variables indicating significant associations with AKI in univariable logistic regression models (sex, clinical classification, IL-2R, IL-10, TNF-α, PCT, lymphocyte, NT-proBNP, hs-cTnI, eGFR, coagulation disorder, D-dimer) were the candidates of multivariable models. To avoid overfitting in the model and interaction effects between observed variables, six variables (COVID-19 clinical classification, lymphocyte percentage, procalcitonin, eGFR, coagulation disorder, D-dimer) were chosen for multivariable analysis. [method = forward stepwise (LR), probability for entry stepwise = 0.05, probability for removal stepwise = 0.10, classification cutoff = 0.5, maximum iteration = 50, include constant in model]. Data were analyzed using R statistical software (v.3.5.2; R Foundation for Statistical Computing, Vienna, Austria; https://www.r-project.org) and IBM SPSS Statistics for Windows version 23.0 (IBM Corporation, Armonk, NY, USA). A two-sided *p* < 0.05 was considered statistically significant. The data in this study are available from the corresponding author.

## Results

### Baseline Characteristics

**Figure 1:**
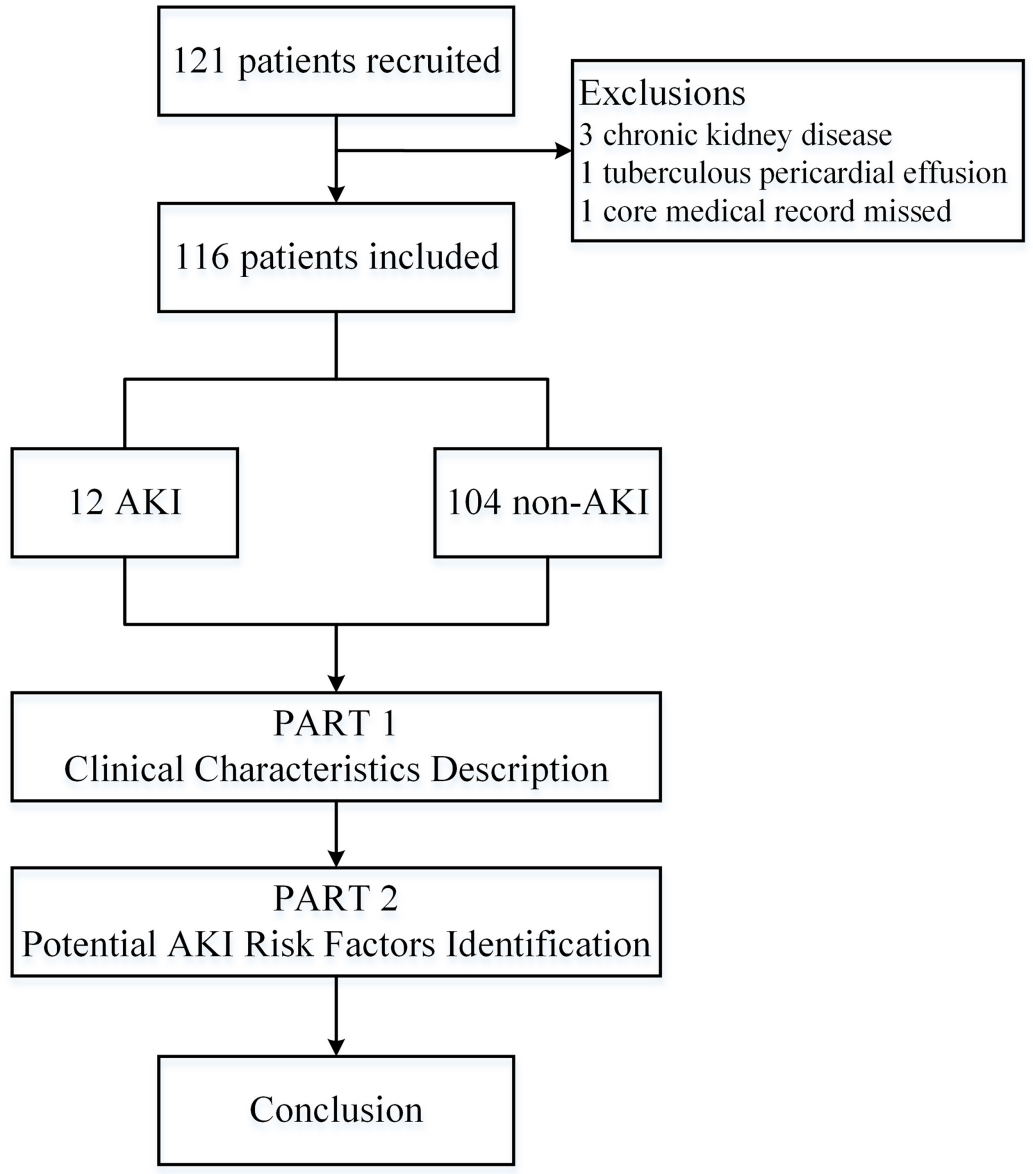
Flowchart of the study. AKI, acute kidney injury.

We pooled 121 in-hospital patients between Mar 9, 2020, and Mar 17, 2020, and 116 eligible patients were included in this study. Five patients were excluded [three cases with chronic kidney disease (one case receiving regular maintenance dialysis, one with diabetic nephropathy, one with nephrotic syndrome), one case diagnosed with tuberculous pericardial effusion after admission, one case missing the core medical record] (Figure 1). Of total patients, the median age was 62.0 (55.0, 69.0) years, exposure history was found in 40 (34.5%) cases, and the dominant onset symptoms were fever [93, (81.9%)] and cough [70, (60.3%)]. Between the two groups, the sex and COVID-19 clinical classification were significantly different, more male and critical cases were in the AKI group. The epidemiological, demographic, and clinical characteristics are presented in Table 1.

**Table 1:** Demographic, epidemiological, and clinical characteristics of the patients.

	**Total, *n =* 116**	**Non-AKI, *n* = 104**	**AKI, *n =* 12**	***p*-value**
Age (years)	62.0 (55.0, 69.0), *n =* 116	62.0 (55.0, 69.0), *n =* 104	66.0 (56.8, 73.0), *n =* 12	0.386
<40	9 (7.8%)	9 (8.7%)	0 (0.0%)	0.595
40–49	5 (4.3%)	3 (2.9%)	2 (16.7%)	0.083
50–59	32 (27.6%)	30 (28.8%)	2 (16.7%)	0.58
60–69	42 (36.2%)	38 (36.5%)	4 (33.3%)	–
70–79	19 (16.4%)	15 (14.4%)	4 (33.3%)	0.206
>80	9 (7.8%)	9 (8.7%)	0 (0.0%)	0.595
**Sex**				
Male	62 (53.4%)	52 (50.0%)	10 (83.3%)	0.028
Female	54 (46.6%)	52 (50.0%)	2 (16.7%)	
Exposure history	40 (34.5%)	35 (33.7%)	5 (41.7%)	0.816
**Comorbidity**				
Any	62 (53_._4%)	53 (51.0%)	9 (75.0%)	0.114
Hypertension	47 (40.5%)	40 (38.5%)	7 (58.3%)	0.309
Diabetes	20 (17.2%)	17 (16.3%)	3 (25.0%)	0.728
CHD	12 (10.3%)	9 (8.7%)	3 (25.0%)	0.208
CD	7 (6.0%)	7 (6.7%)	0 (0.0%)	–
Malignancy	10 (8.6%)	9 (8.7%)	1 (8.3%)	–
Hyperuricemia	2 (1.7%)	1 (1.0%)	1 (8.3%)	0.197
Hypothyroidism	3 (2.6%)	1 (1.0%)	2 (16.7%)	0.028
COPD	3 (2.6%)	2 (1.9%)	1 (8.3%)	0.282
Other	9 (7.8%)^*^	9 (8.7%)^*^	0 (0.0%)	0.595
Fever	93 (80.2%)	81 (77.9%)	12 (100.0%)	0.151
Cough	70 (60.3%)	64 (61.5%)	6 (50.0%)	0.644
Sputum	30 (25.9%)	28 (26.9%)	2 (16.7%)	0.674
Chest distress	17 (14.7%)	13 (12.5%)	4 (33.3%)	0.133
Dyspnea	28 (24.1%)	22 (21.1%)	6 (50.0%)	0.064
Headache	8 (6.9%)	8 (7.7%)	0 (0.0%)	–
Myalgia	11 (9.5%)	9 (8.7%)	2 (16.7%)	0.318
Fatigue	38 (32.8%)	33 (31.7%)	5 (41.7%)	0.712
Hypersomnia	2 (1.7%)	1 (1.0%)	1 (8.3%)	0.197
Nausea	7 (6.0%)	5 (4.8%)	2 (16.7%)	0.154
Anorexia	15 (12.9%)	13 (12.5%)	2 (16.7%)	–
Diarrhea	27 (23.3%)	26 (25.0%)	1 (8.3%)	0.351
Asymptomatic	3 (2.6%)	3 (2.9%)	0 (0.0%)	–
Body temperature (°C)	37.8 (36.7, 38.5)	37.7 (36.6, 38.5)	37.9 (36.8,38.5)	0.72
≤37.3	44 (37.9%)	40 (38.5%)	4 (33.3%)	0.974
37.4–38	32 (27.6%)	27 (26.0%)	5 (41.7%)	0.417
38.1–39	35 (30.2%)	33 (31.7%)	2 (16.7%)	0.457
≥39.1	5 (4.3%)	4 (3.8%)	1 (8.3%)	0.427
SBP (mmHg)	132.0 (120.0, 141.0), *n =* 116	130.0 (120.0, 141.0), *n =* 104	137.5 (131.3, 148.8), *n =* 12	0.121
DBP (mmHg)	80.5 (75.0, 90.0), *n =* 116	80.0 (74.3, 90.0), *n =* 104	84.0 (76.3, 90.0), *n =* 12	0.43
Respiratory rate (bpm)	20.0 (20.0, 24.0), *n =* 116	20.0 (20.0, 22.8), *n =* 104	20.5 (20.0, 27.5), *n =* 12	0.302
>24	30 (25.9%)	25 (24.0%)	5 (41.7%)	0.331
Pulse (bpm)	91.0 (80.0, 105.0), *n =* 116	90.0 (80.0, 105.0), *n =* 104	97.5 (87.0, 117.8), *n =* 12	0.149
**Type of diagnosis**				
Clinical diagnosis	39 (33.6%)	35 (33.7%)	4 (33.3%)	–
Laboratory diagnosis	77 (66.4%)	69 (66.3%)	8 (66.7%)	–
Clinical classification				–
Noncritical	93 (80.2%)	88 (84.6%)	5 (41.7%)	0.002
Critical	23 (19.8%)	16 (15.4%)	7 (58.3%)	

* *Others including coma, pulmonary tuberculosis, benign prostatic hyperplasia, hemoptysis, hepatitis B, hyperlipidemia, anemia, chronic gastric, asthma. Continuous variables were presented as median (IQR). Categorical variables were presented as n (%), and percentages do not total 100% owing to missing data. AKI, acute kidney injury; CHD, coronary heart disease; CD, cerebrovascular disease; COPD, chronic obstructive pulmonary disease; SBP, systolic blood pressure; DBP, diastolic blood pressure*.

### Laboratory and Radiographic Findings on Admission

The inflammatory indicators, interleukin-2R [553.50 (319.25, 897.75) U/mL], interleukin-6 [15.57 (3.07, 39.62) pg/mL], tumor necrosis factor-α [8.40 (5.95, 11.75) pg/mL], procalcitonin [0.06 (0.03, 0.15) ng/mL], and high-sensitivity C-reactive protein [40.10 (12.65, 124.10) mg/L], were significantly higher than the normal range and higher in the AKI group than in the non-AKI group (*p* = 0.006, *p* = 0.017, *p* = 0.002, *p* < 0.0001, *p* = 0.035). The findings of the blood routine test on admission showed decreased lymphocyte [percentage 14.25 (7.83, 23.23)%, count 0.83 (0.53, 1.34) × 10^9^/L] and increased neutrophil [percentage 78.40 (64.68, 86.55)%, count 4.82 (3.42, 7.53) × 10^9^/L]. N-terminal pro-brain natriuretic peptide (NT-proBNP), high-sensitivity cardiac troponin I (hs-cTnI), prothrombin time (PT), activated partial thromboplastin time (APTT), and D-dimer level on admission were also found significantly higher in the AKI group (*p* = 0.005, *p* = 0.005, *p* = 0.001, *p* = 0.003, *p* = 0.005). Laboratory and radiographic findings of patients on admission are summarized in Table 2.

**Table 2:** Laboratory and radiographic findings of the patients on admission.

	**Total, *n =* 116**	**Non-AKI, *n =* 104**	**AKI, *n =* 12**	***p*-value**	**Normal range**
SARS-CoV-2 IgM (AU/mL)	49.34 (13.96, 106.47), *n =* 82	50.8 (15.2, 106.5), *n =* 78	28.00 (1.90, 199.23), *n =* 4	0.451	
SARS-CoV-2 IgG (AU/mL)	169.76 (108.52, 190.63), *n =* 82	169.99 (109.44, 191.71), *n =* 78	129.35 (26.59, 183.39), *n =* 4	0.401	≤10
SARS-CoV-2 IgG/IgM	2.82 (1.29, 7.11), *n =* 82	2.81 (1.19, 6.21), *n =* 78	8.36 (1.47, 21.32), *n =* 4	0.301	≤10
IL-1β, ≥5 (pg/mL)	39 (33.6%)	34 (32.7%)	5 (41.7%)	0.764	<5
IL-2R (U/mL)	553.50 (319.25, 897.75), *n =* 92	537.00 (316.00, 861.00), *n =* 82	1172.50 (715.00, 2285.75), *n =* 10	0.006	223–710
<710	36 (31.0%)	28 (26.9%)	8 (66.7%)	0.013	
IL-6 (pg/mL)	15.57 (3.07, 39.62), *n =* 96	11.61 (2.71, 37.82), *n =* 86	32.85 (20.15, 401.90), *n =* 10	0.017	<7
<7	63 (54.3%)	54 (51.9%)	9 (75.0%)	0.129	
IL-8 (pg/mL)	15.95 (7.98, 43.75), *n =* 90	14.40 (7.55, 42.00), *n =* 80	22.45 (16.08, 62.13), *n =* 10	0.284	<62
IL-10, ≥9.1 (pg/mL)	22 (19.0%)	17 (16.3%)	5 (41.7%)	0.084	<9.1
TNF-α (pg/mL)	8.40 (5.95, 11.75), *n =* 93	8.20 (5.78, 10.53), *n =* 82	15.70 (9.20, 19.10), *n =* 11	0.002	<8.1
<8.1	52 (44.8%)	42 (40.4%)	10 (83.3%)	0.005	
PCT (ng/mL)	0.06 (0.03, 0.15), *n =* 97	0.05 (0.03, 0.12), *n =* 85	1.08 (0.08, 1.78), *n =* 12	<0.0001	0.02–0.05
<0.05	57 (49.1%)	57 (54.8%)	0 (0.0%)	<0.0001	
0.05–0.1	27 (23.3%)	23 (22.1%)	4 (33.3%)	0.61	
>0.1	32 (27.6%)	24 (23.1%)	8 (66.7%)	0.004	
hs-CRP (mg/L)	40.10 (12.65, 124.10), *n =* 92	36.10 (9.70, 116.30), *n =* 81	105.50 (39.10, 174.00), *n =* 11	0.035	<1
≥1	85 (73.3%)	74 (71.2%)	11 (91.7%)	0.24	
ESR (mm/H)	32.0 (17.0, 65.5), *n =* 52	28.0 (15.3, 58.0), *n =* 46	55.5 (31.0, 84.8), *n =* 6	0.094	2–20
Elevated	33 (28.4%)	27 (25.0%)	6 (50.0%)	0.159	
Leukocyte count (× 10^9^ per L)	6.73 (4.93, 9.92), *n =* 110	6.55 (4.87, 9.37), *n =* 99	12.73 (5.16, 19.20), *n =* 11	0.043	3.5–9.5*10^9^
Neutrophil percentage	78.40 (64.68, 86.55), *n =* 110	75.60 (64.10, 86.20), *n =* 99	83.20 (76.30, 93.30), *n =* 11	0.041	40–75
Neutrophil count (× 10^9^ per L)	4.82 (3.42, 7.53), *n =* 104	4.75 (3.37, 7.07), *n =* 95	6.85 (3.75, 14.99), *n =* 9	0.087	1.8–6.3*10^9^
Lymphocyte percentage	14.25 (7.83, 23.23), *n =* 108	14.70 (8.23, 24.58), *n =* 96	7.50 (3.58, 11.13), *n =* 12	0.003	20–50
Lymphocyte count (× 10^9^ per L)	0.83 (0.53, 1.34), *n =* 107	0.91 (0.54, 1.35), *n =* 96	0.59 (0.42, 0.83), *n =* 11	0.1	1.1–3.2*10^9^
Erythrocyte count, (× 10^12^ per L)	4.11 (3.63, 4.51), *n =* 104	4.14 (3.69, 4.51), *n =* 96	3.75 (2.73, 4.65), *n =* 8	0.487	F 3.8–5.1 (M 4.3–5.8)*10^12^
Hemoglobin (g/L)	125.0 (108.0, 134.5), *n =* 109	127.0 (110.0, 134.5), *n =* 97	106.0 (83.8, 137.8), *n =* 12	0.089	F 115–150 (M 130–175)
Platelet count (× 10^9^ per L)	196.00 (157.25, 261.00), *n =* 102	199.50 (158.50, 269.00), *n =* 92	155.50 (121.00, 197.50), *n =* 10	0.021	125–350*10^9^
<125	12 (10.3%)	9 (8.7%)	3 (25.0%)	0.208	
Glucose (μmol/L)	6.57 (5.50, 8.74), *n =* 102	6.57 (5.37, 8.73), *n =* 91	6.41 (5.80, 9.19), *n =* 11	0.608	4.11–6.05
Ferroprotein (μg/L)	570.8 (276.5, 1241.0), *n =* 81	542.0 (268.0, 1199.5), *n =* 74	1264.0 (416.0, 1652.0), *n =* 7	0.168	F 15–150 (M 30–400)
ALT (U/L)	27.0 (18.0, 44.0), *n =* 106	28.0 (18.5, 44.0), *n =* 97	23.0 (15.5, 38.5), *n =* 9	0.411	F <33 (M <41)
AST (U/L)	27.0 (21.0, 39.0), *n =* 108	27.0 (21.0, 38.0), *n =* 97	26.7 (21.0, 48.0), *n =* 11	0.655	F <32 (M <40)
ALP (U/L)	71.0 (59.0, 91.3), *n =* 102	71.0 (57.5, 89.0), *n =* 92	72.0 (62.8, 171.5), *n =* 10	0.251	F 35–105 (M 40–130)
LDH (U/L)	302.0 (220.0, 403.0), *n =* 103	291.5 (212.3, 400.0), *n =* 92	356.0 (311.0, 423.0), *n =* 11	0.051	F 135–214 (M 135–225)
Albumin (g/L)	33.75 (29.98, 39.15), *n =* 110	34.20 (30.90, 39.40), *n =* 99	28.30 (25.70, 33.80), *n =* 11	0.013	35–52
≤25	14 (12.1%)	10 (9.6%)	4 (33.3%)	0.055	
Globulin (g/L)	33.4 (30.1, 36.8), *n =* 96	33.1 (30.4, 36.4), *n =* 88	35.9 (27.8, 42.7), *n =* 8	0.533	20–35
Total bilirubin (μmol/L)	9.55 (6.93, 14.25), *n =* 102	9.60 (6.85, 14.45), *n =* 93	8.30 (6.95, 13.75), *n =* 9	0.728	F <21 (M <26)
Total cholesterol (mmol/L)	3.65 (3.12, 4.34), *n =* 95	3.65 (3.13, 4.43), *n =* 89	3.65 (2.68, 4.63), *n =* 6	0.68	<5.18
NT-proBNP	251.0 (64.0, 867.3), *n =* 82	220.5 (49.5, 683.3), *n =* 72	1250.5 (254.5, 3072.8), *n =* 10	0.005	–
Elevated	29 (25.0%)	22 (21.2%)	7 (58.3%)	0.014	
hs-cTnI (pg/mL)	11.15 (5.50, 24.72), *n =* 74	10.90 (4.80, 19.50), *n =* 63	78.40 (8.90, 241.90), *n =* 11	0.005	F ≤15.6 (M 34.2)
Elevated	20 (17.2%)	14 (13.5%)	6 (50.0%)	0.006	
PT (s)	14.3 (13.5, 15.1), *n =* 104	14.1 (13.5, 14.9), *n =* 92	15.5 (14.5, 16.2), *n =* 12	0.001	11.5–14.5
APPT (s)	40.1 (36.2, 45.0), *n =* 93	39.7 (35.7, 44.4), *n =* 83	45.8 (42.4, 52.9), *n =* 10	0.003	29.0–42.0
Coagulation disorder	19 (16.4%)	14 (13.5%)	5 (41.7%)	0.037	
D-dimer (μg/mL)	1.31 (0.49, 4.31), *n =* 106	1.25 (0.48, 3.18), *n =* 94	4.80 (1.47, 18.34), *n =* 12	0.005	<0.5
Ground-glass opacity	23 (19.8%)	20 (19.2%)	3 (25.0%)	0.926	
Patchy shadow	6 (5.2%)	5 (4.8%)	1 (8.3%)	0.489	
Infection indicated	87 (7.5%)	79 (76.0%)	8 (66.7%)	0.725	

*Continuous variables were presented as median (IQR) and the number of cases available was noted. Categorical variables were presented as n (%), and percentages do not total 100% owing to missing data. AKI, acute kidney injury; IL, interleukin; TNF-α, tumor necrosis factor-α; PCT, procalcitonin; hs-CRP, high-sensitivity C-reactive protein; ESR, erythrocyte sedimentation rate; ALT, alanine aminotransferase; AST, aspartate aminotransferase; ALP, alkaline phosphatase; LDH, lactic dehydrogenase; NT-proBNP, N-terminal pro-brain natriuretic peptide; hs-cTnI, high-sensitivity cardiac troponin I; PT, prothrombin time; APTT, activated partial thromboplastin time*.

### Treatments and Outcomes

Of the entire series, antibiotics were administered in 97 (83.6%) cases, corticosteroids were administered in 63 (54.3%) cases, and 106 (91.4%) received antiviral treatment (including 36 cases accepting Chinese traditional medicine). As of Mar 17, the total observation period was 28.0 (13.5, 41.0) days, while 108 (93.1%) patients were still in hospital. Among them, 19 patients were treated in the ICU and 89 in the general ward. The treatments and outcomes were compared between the sCr elevated group and sCr normal group and summarized in Table S1.

### Kidney Injury and Associated Risk Factors

The dynamic profiles of sCr and eGFR of all patients, the sCr elevated group, and the sCr normal group are shown in Figure S1. On admission, 21 (18.1%) were observed with elevated sCr. Hematuria was observed in 24 (20.7%) patients [(+) in 16 (13.8%) cases; (++ to +++) in 8 (6.9%)]. Proteinuria was observed in 25 (21.6%) patients [(+) in 19 (16.4) cases; (++ to +++) in 6 (5.2%) cases] (Table 3).

**Table 3:** Laboratory findings of the kidney function of the patients on admission.

	**Total, *n =* 116**	**Non-AKI, *n =* 104**	**AKI, *n =* 12**	***p*-value**	**Normal range**
Potassium (mmol/L)	4.21 (3.81, 4.53), *n =* 98	4.20 (3.81, 4.52), *n =* 89	4.37 (3.86, 5.33), *n =* 9	0.449	3.5–5.10
Sodium (mmol/L)	139.8 (136.0, 141.7), *n =* 99	139.9 (136.9, 141.6), *n =* 91	135.6 (130.2, 143.0), *n =* 8	0.372	136–145
sCr (μmol/L)	67.5 (55.0, 82.0), *n =* 114	66.0 (54.0, 79.3), *n =* 102	127.0 (66.3, 166.5), *n =* 12	<0.0001	F 45–84 (M 59–104)
eGFR (mL/min/1.73 m^2^)	95.52 (79.56, 117.04), *n =* 114	98.95 (82.02, 117.93), *n =* 102	53.49 (38.62, 87.78), *n =* 12	<0.0001	>90
<60	7 (6.0%)	3 (2.9%)	4 (33.3%)	0.002	
BUN (mmol/L)	5.10 (3.90, 6.37), *n =* 108	4.80 (3.55, 6.25), *n =* 97	8.70 (5.80, 14.70), *n =* 11	<0.0001	F 3.1–8.8 (M 1.7–8.3)
Hematuria					–
Any positive	24 (20.7%)	20 (19.2%)	4 (33.3%)	0.444	
+	16 (13.8%)	13 (12.5%)	3 (25.0%)	0.455	
++ ~ +++	8 (6.9%)	7 (6.7%)	1 (8.3%)	0.595	
Urinary leukocyte					–
Any positive	18 (15.5%)	16 (15.4%)	2 (16.7%)	–	
+	8 (6.9%)	7 (6.7%)	1 (8.3%)	0.595	
++ ~ +++	10 (8.6%)	9 (8.7%)	1 (8.3%)	–	
Proteinuria					–
Any positive	25 (21.6%)	19 (18.3%)	6 (50.0%)	0.031	
+	19 (16.4%)	16 (15.5%)	3 (25.0%)	0.66	
++ ~ +++	6 (5.2%)	3 (2.9%)	3 (25.0%)	0.014	

*Continuous variables were presented as median (IQR), and the number of cases available was noted. Categorical variables were presented as n (%), and percentages do not total 100% owing to missing data. BUN, blood urea nitrogen; sCr, serum creatinine; eGFR, estimated glomerular filtration rate*.

Over the observation period, 12 (10.3%) patients met the diagnostic criteria of AKI [9 (7.8%) of stage 1 and 3 (2.6%) of stage 2)], including 7 (6.0%) patients of the sCr elevated group. Of the sCr normal group, AKI were developed in 5 (4.3%) patients [3 (2.6%) of stage 1 and 2 (1.7%) of stage 2], recorded as in-hospital AKI. Four (80.0%) of five in-hospital AKI occurring after the patient's health status worsened. A reduction in oxygen saturation was observed, while two patients developed respiratory failure and two acute respiratory distress syndrome. One of the five in-hospital AKI was recognized as a result of the side effects of the antivirus drug. Renal function recovered was observed in four patients, while one patient received continuous renal replacement therapy (CRRT) (Tables S2, S3).

In univariable analysis, sex, clinical classification, IL-2R, IL-10, TNF-α, PCT, neutrophil percentage, lymphocyte percentage, NT-proBNP, hs-cTnI, eGFR, coagulation disorder, and D-dimer were associated with AKI (Table 4). Multivariable regression showed increasing odds of COVID-19-related AKI associated with COVID-19 clinical classification (OR = 8.155, 95% CI = 1.848–35.983, ref = non-critical, *p* = 0.06), procalcitonin more than 0.1 ng/mL (OR = 4.822, 95% CI = 1.095–21.228, *p* = 0.037), and eGFR <60 mL/min/1.73 m^2^ (OR = 13.451, 95% CI = 1.617–111.891, *p* = 0.016) (Table 4).

**Table 4:** Risk factors associated with COVID-19-related AKI.

	**Univariable**				**Multivariable**			
	***p*-value**	**OR**	**95% CI for OR**		***p*-value**	**OR**	**95% CI for OR**	
			**Lower limit**	**Upper limit**			**Lower limit**	**Upper limit**
Age, per 1 year increased, *n =* 116	0.634	1.012	0.964	1.063	–	–	–	–
Sex, ref = female, *n =* 116	0.044	5.000	1.044	23.939	–		–	–
Comorbidity present, ref = not present, *n =* 116	0.127	2.887	0.739	11.270	–	–	–	–
Clinical classification, ref = non-critical, *n =* 116	0.002	7.700	2.173	27.288	0.006	8.155	1.848	35.983
IL-2R, ref = <710 U/mL, *n =* 116	0.009	5.429	1.515	19.448	–	–	–	–
IL-10, ref = <9.1 pg/mL, *n =* 116	0.044	3.655	1.037	12.885	–	–	–	–
TNF-α, ref = <8.1 pg/mL, *n =* 116	0.012	7.381	1.539	35.403	–	–	–	–
PCT, ref = <0.1 ng/mL, *n =* 116	0.004	6.667	1.846	24.073	0.037	4.822	1.095	21.228
Neutrophil, per 1% increased, *n =* 110	0.043	1.068	1.002	1.138	–	–	–	–
Lymphocyte, per 1% increased, *n =* 108	0.010	0.859	0.766	0.964	–	–	–	–
Hemoglobin, per 1 g/L increased, *n =* 109	0.068	0.977	0.953	1.002	–	–	–	–
LDH, ref = >214 U/L, *n =* 116	0.059	7.452	0.927	59.900	–	–	–	–
Albumin, ref = >25 g/L, *n =* 116	0.083	5.000	0.812	30.785	–	–	–	–
Total cholesterol, ref = >5.18 mmol/L, *n =* 116	0.970	0.960	0.111	8.306	–	–	–	–
NT-proBNP elevated, ref = normal, *n =* 116	0.009	5.218	1.509	18.039	–	–	–	–
hs-cTnI elevated, ref = normal, *n =* 116	0.004	6.429	1.816	22.753	–	–	–	–
eGFR, ref = <60 mL/min/1.73 m^2^, *n =* 116	0.001	16.833	3.197	88.624	0.016	13.451	1.617	111.891
Coagulation disorder, ref = normal, *n =* 116	0.019	4.592	1.279	16.488	–	–	–	–
D-dimer, ref = >1 μg/mL, *n =* 116	0.029	10.185	1.269	81.767	–	–	–	–

*Multivariable model performance (area under curve [AUC], Hosmer and Lemeshow goodness-of-fit test): AUC 0.849 (95% CI 0.729–0.968), χ^2^ = 1.231, p = 0.540. Continuous variables were per 1 unit increased. IL, interleukin; TNF-α, tumor necrosis factor-α; PCT, procalcitonin; hSCRP, high-sensitivity C-reactive protein; ALT, alanine aminotransferase; AST, aspartate aminotransferase; ALP, alkaline phosphatase; LDH, lactic dehydrogenase; PT, prothrombin time; APTT, activated partial thromboplastin time. NT-proBNP, N-terminal pro-brain natriuretic peptide; hs-cTnI, high-sensitivity cardiac troponin I*.

## Discussion

The present retrospective cohort study identified several risk factors for COVID-19-related AKI. In particular, COVID-19 clinical classification, procalcitonin more than 0.1 ng/mL, and eGFR <60 mL/min/1.73 m^2^. Additionally, in univariable analysis, sex, IL-2R, IL-10, TNF-α, neutrophil percentage, lymphocyte percentage, NT-proBNP, hs-cTnI, coagulation disorder, and D-dimer were associated with kidney injury.

Reviewing the previous published literature on COVID-19 (summarized in Table 5), patients with COVID-19 presented varying degrees of renal injury, with incidence varies from 0.5 to 28.8%. The incidence of AKI was lower among mild COVID-19 cases, while that of severe or critical cases was increased (10, 14, 17–25). In the present study, five (71.4%) patients with AKI of the sCr elevated group were diagnosed as the critical cases on admission, and four (80.0%) of five in-hospital AKI occurring after the condition worsened (as two developing respiratory failure and two acute respiratory distress syndrome). We found increasing odds of COVID-19-related kidney injury associated with the COVID-19 clinical classification. In the Chinese management guideline for COVID-19 (trial version 7.0) (12), oxygen saturation is the key parameter of clinical classification. Moreover, decreased oxygen saturation was observed in patients with AKI in the present study as well. Patients with COVID-19 may suffer kidney damage due to hypoxia. During acute hypoxemia, adenosine levels increase as adenosine triphosphate (ATP) hydrolysis exceeds synthesis resulting in intrarenal vasoconstriction, decreased renal perfusion, and drop of glomerular filtration rate (26). What is more, we also observed that eGFR decreased in COVID-19 patients resulting from heart failure. Based on our data, eGFR <60 mL/min/1.73 m^2^ was proven to be significant.

**Table 5:** Review of the COVID-19 related kidney impairment.

	***N***	**Pre-existing renal conditions**	**COVID-19-related kidney impairment**	**Notes**
Li et al. (10)	59	–	–	32/51 exhibited proteinuria, Cre elevated in 11/59, Bun elevated in 16/59; 27/27 exhibited radiographic abnormalities of the kidneys
Cheng et al. (14)	710	–	22 AKI	On admission, sCr elevated in 110/710, 44% have proteinuria hematuria and 26.9% have hematuria; mortality of inpatients with elevated baselines Cr was 30.9%
Yang et al. (17)	52	–	15 AKI	sCr elevated significantly in survivors
Xu et al. (18)	62	1	–	sCr elevated in 2/62 of time since symptom onset ≤10 days group
Wang et al. (19)	138	4	5 AKI	Bun and sCr elevated significantly in ICU group (vs. non-ICU group)
Shi et al. (20)	81	–	3 CRF	–
Huang et al. (21)	41	–	3 AKI	sCr elevated in 4/41 (2/41 of ICU group, 2/41 of non-ICU group)
Guan et al. (22)	1,099	8	6 AKI	sCr elevated in 12/752 (16/614 of non-severe group, 6/138 of severe group)
Chen et al. (23)	99	–	3 AKI	Bun increased in 6/99, decreased in 17/99; sCr increased in 3/99, decreased in 21/99
Chan et al. (24)	7	–	–	Bun elevated in 2/7
Diao et al. (25)	85	5	23 ARF	Elder patients (≥60 years old) were easier to develop ARF; perished cases have a rapid decrease of eGFR but quickly boosting ccr and bun; H&E staining: severe acute tubular necrosis; immunohistochemistry: SARS-CoV-2 NP antigen accumulated in kidney tubules.
Our study	116	–^*^	12 AKI	sCr elevated in 21/116, 12(10.3%) AKI (5 in-hospital AKI); increasing odds of AKI associated with COVID-19 clinical classification, procalcitonin more than 0.1 ng/mL, and eGFR <60 mL/min/1.73 m^2^

* *Three patients excluded from the study, as one case receiving regular maintenance dialysis, one diabetic nephropathy, one nephrotic syndrome. AKI, acute kidney injury; ICU, intensive care unit; Bun, blood urea nitrogen; sCr, serum creatinine; Cre, plasma creatinine; CRF, chronic renal failure; ARF, acute renal failure; eGFR, estimated glomerular filtration rate; SARS-CoV-2, severe acute respiratory syndrome coronavirus 2; NP, nucleocapsid protein; H&E staining and hematoxylin–eosin staining*.

Previous studies had discussed the role of cytokine storm in the pathogenesis of severe viral pneumonia caused by SARS-CoV (27). Virus-induced cytokines might exert indirect effects on renal tissue. The autopsies of COVID-19 suggested cytokine storms in patients (28). In the present study, IL-2R, IL-6, TNF-α, and hs-CRP were significantly higher than the normal range and higher in the AKI group, and PCT more than 0.1 ng/mL was found to be associated with COVID-19-related AKI. According to a recent meta-analysis study, serial PCT measurement may play a role for predicting evolution toward a more severe form of disease (29).

Impaired renal function could recover, receiving clinical intervention, including active antiviral treatment, vasoactive agent for increasing renal perfusion, and advanced respiratory support for improving oxygen saturation. It is necessary to timely apply renal replacement therapy or blood purification in severe cases with COVID-19, complicated with AKI, systemic inflammatory response syndrome (SIRS), or multiple-organ dysfunction syndromes (MODS). Blood purification techniques, including plasma exchange, adsorption, perfusion, and filtration, in particular CRRT, played an important role in the rescue and treatment of severe acute respiratory syndromes (SARS), Middle East Respiratory Syndrome (MERS), and other sepsis (30, 31). The efficacy and safety of antiviral drugs for COVID-19 require further confirmation by clinical experiments, and clinicians should thus be aware of the potential dosage adjustments and renal adverse events (32).

As far as we know, the present study was the first to identify the potential risk factors of COVID-19-related AKI and providing treatment experience for the clinical management of COVID-19-related AKI. Our study has some limitations. Due to the retrospective study design, not all needed laboratory findings were collected, such as urine routine test and SARS-CoV-2 antibody. Therefore, their role might be underestimated. The sample size might limit the interpretation of our findings. Considering the total number of AKI (*n* = 12) in our study and overfitting in the model are almost inevitable. The sample size of this study does not meet the requirements of EPV (event per variable), so the results may not be robust. However, such patients are rare and the results are somewhat interpretable, we still present the results of the study. The findings should be interpreted as exploratory and descriptive; therefore, a multicenter, large sample size, and prospective study will be required. Continued observations of the natural history of the COVID-19-related kidney injury are needed.

## Conclusion

Patients with COVID-19 presented varying degrees of kidney injury, and the AKI was likely to be related to multiorgan failure rather than the kidney tropism of SARS-CoV-2. The potential risk factors of COVID-19 clinical classification, eGFR, and procalcitonin could help clinicians to identify patients with kidney injury at an early stage.

## Data Availability Statement

The raw data supporting the conclusions of this article will be made available by the authors, without undue reservation.

## Ethics Statement

This study protocol and written informed consent were approved by the Medical Ethics Committee (No. TJ-C20200155) and COVID-19 Academic Committee of Tongji Hospital.

## Author Contributions

XZ, JW, and ZW conceived and designed the study. JW did the analyses and prepared the tables and figures with XL, YZ, ZW, CL, XW, and KY. JW wrote the original draft of the manuscript with HL, XY, CF, YW, SL, and XZ. All authors reviewed and approved the manuscript before submitting it for publication.

## Conflict of Interest

The authors declare that the research was conducted in the absence of any commercial or financial relationships that could be construed as a potential conflict of interest.
