# Supplementary material for: Identify the Risk Factors of COVID-19-Related Acute Kidney Injury: A Single-Center, Retrospective Cohort Study
Source: Front Med (Lausanne). 2020 Jul 28;7:436. doi: 10.3389/fmed.2020.00436 (PMC7399623; doi:10.3389/fmed.2020.00436)
Supplement: Supplementary file 1 [file Data_Sheet_1.PDF]

Supplementary Material

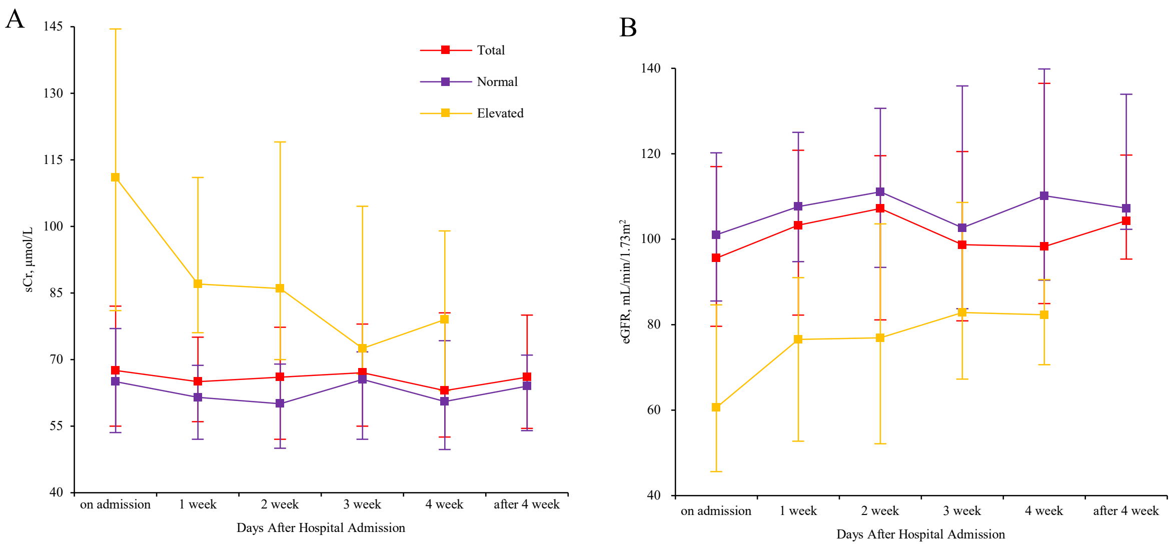

FIGURE S1 | Dynamic profile of kidney function. (A) Dynamic profile of sCr; (B) Dynamic profile of eGFR. sCr, serum creatinine; eGFR, estimated glomerular filtration rate. The data of sCr and eGFR after 4 week in sCr elevated group was missing.

TABLE S1 | Treatments and outcomes by the deadline.

	Total n=116	sCr normal n=95	sCr elevated n=21	p value
**Treatments**				
Antibiotics	97(83.6%)	80(84.2%)	17(81.0%)	0.969
Antiviral treatment	106(91.4%)	87(91.6%)	19(90.5%)	-
Antifungal treatment	10(8.6%)	5(5.3%)	5(23.8%)	0.021
Corticosteroids	63(54.3%)	47(49.5%)	16(76.2%)	0.026
Intravenous immunoglobin	21(18.1%)	17(17.9%)	4(19.0%)	-
Blood transfusion	11(9.5%)	6(6.3%)	5(23.8%)	0.039
Chinese traditional medicine	36(31.0%)	30(31.6%)	6(28.6%)	0.787
Nasal cannula oxygen inhalation	55(47.4%)	46(48.4%)	9(42.9%)	0.644
Non-invasive mechanical ventilation	5(4.3%)	5(5.3%)	0(0.0%)	0.583
Invasive mechanical ventilation	20(17.2%)	11(11.6%)	9(42.9%)	0.002
Trachea intubation	11(9.5%)	5(5.3%)	6(28.6%)	0.004
Tracheostomy tube	5(4.3%)	5(5.3%)	0(0.0%)	0.583
ECMO	4(3.4%)	1(1.1%)	3(14.3%)	0.019
CRRT	5(4.3%)	1(1.1%)	4(19.0%)	0.004
Convalescent plasma	3(2.6%)	3(3.2%)	0(0.0%)	-
Thoracic drainage	2(1.7%)	1(1.1%)	1(4.8%)	0.331
Bronchoalveolar lavage	2(1.7%)	1(1.1%)	1(4.8%)	0.331
Hemoperfusion	2(1.7%)	1(1.1%)	1(1.1%)	0.331
**Outcomes***				
In the ICU	19(16.4%)	12(12.6%)	7(33.3%)	0.046
In general ward	89(76.7%)	76(80.0%)	13(61.9%)	0.136
Discharge	8(6.9%)	7(7.4%)	1(4.8%)	-
Secondary infection	34(29.3%)	21(22.1%)	13(61.9%)	＜0.0001
Respiratory failure	25(21.6%)	15(15.8%)	10(47.6%)	0.004
ARDS	31(26.7%)	21(22.1%)	10(47.6%)	0.017
Heart failure	6(5.2%)	1(1.1%)	5(23.8%)	＜0.0001
Alimentary tract hemorrhage	3(2.6%)	0(0.0%)	3(14.3%)	0.005
Liver damage	4(3.4%)	3(3.2%)	1(4.8%)	0.555
AKI	12(10.3%)	5(5.3%)	7(33.3%)	0.001
Stage 1	9(7.8%)	3(3.2%)	6(28.6)	0.001
Stage 2	3(2.6%)）	2(2.2%)	1(4.8%)	0.454
Anemia	7(6.0%)	3(3.2%)	4(19.0%)	0.024
Inflammatory factor storm	3(2.6%)	1(1.1%)	2(9.5%)	0.084
Shock	2(1.7%)	2(2.1%)	0(0.0%)	-
Other	8(6.9%)	5(5.3%)[a]	3(14.3%)[b]	0.317

*The total observe period was 28.0 (13.5, 41.0) days. [a]Includes: Cardiac arrest & hypoxic, encephalopathy, gross hematuria, edema lower extremity, drug-induced leukopenia, drug-inducedrespiratory depression. [b]Includes: gout, delirium, drug-induced leukopenia. sCr, serum creatinine; ECMO, extracorporeal membrane oxygenation; CRRT, continuous renal replacement therapy; ICU, intensive care unit; ARDS, acute respiratory distress syndrome; AKI, acute kidney injury.

TABLE S2 | Review of the clinical management course of the 5 in-hospital AKI

Patient ID	Sex	Age, years	Date of onset	Symptom of onset	Diagnosis on admission	Comorbidity	Treatments after admission	New diagnosis in-hospital	Time from onset to ICU admission, days	Time from onset to AKI, days	Management after AKI
250****341	female	75	2020/2/6	fever	severe	HEN, DAM, CHD	nasal cannula oxygen inhalation, antivirus, antibiotics	hypokalemia, HF, anemia	30	32	dopamine to improve renal perfusion
250****870	female	73	2020/2/7	fever, cough, sputum, fatigue, myalgia	critical	DAM, hypothyroidism	antivirus, antibiotics, corticosteroids, BiPAP → trachea intubation(2/15)	ARF, second infection	37	14	exit the Remdisivir clinical trial
250****193	male	66	2020/1/21	fever	critical	HEN, hypothyroidism	BiPAP → trachea intubation antivirus, antibiotics, corticosteroids	gastrointestinal hemorrhage, RF, second infection	20	48	vasoactive agent (norepinephrine)
250****591	male	56	2020/1/21	fever(Tmax 37.8℃), cough, fatigue	critical	HEN	antivirus, antibiotics, corticosteroids antimycotic, BiPAP→ trachea intubation（2/10 → ECMO（2/11）	ARDS, ARF, MDR, septic shock gastrointestinal hemorrhage	14	32	CRRT
250****542	male	73	2020/1/31	fever(Tmax 38.9℃), cough	general	HTN, DAM	antivirus, antibiotics, corticosteroids; BiPAP	ARDS	5（2/15 out）	10	trachea intubation，IVIG and antibiotics

HEN, hypertension; DAM, diabetes mellitus; BiPAP, Bi-Level positive airway pressure; ECMO, extracorporeal membrane oxygenation; HF, heart failure; ARF, acute respiratory failure; ARDS, acute respiratory distress syndrome; MDR, multi-drug resistance; CRRT, continuous renal replacement therapy; IVIG, intravenous immunogloblin; AKI, acute kidney injury; sCr, serum creatine.

TABLE S3| Laboratory findings of sCr and eGFR of the 5 in-hospital AKI.

Patient ID	Days after admission	0	1	2	3	4	5	6	7	8	9	10	11	12	13	14	15	16	17	18	19	20	21
250****341	sCr, µmol/L	70	-		61	-	-	61		-	-	-	-	-	-	69	-	72	-	-	78	81	-
	eGFR(CKD-EPI), mL/min/1.73m^2	73.3	-		85.4	-	-	86.4	-	-	-	-	-	-	-	74.5	-	70.8	-	-	64.3	67.4	-
250****870	sCr, µmol/L	-	-	-	61	-	58	68	66	70	119	-	-	-	100	94	-	69	57	-	-	56	49
	eGFR(CKD-EPI), mL/min/1.73m^2	-	-	-	86	-	88	77	80	74	39	-	-	-	48	52	-	75	88	-	-	89	93
250****193	sCr, µmol/L	80	-	-	57	-	70	-	-	-	83	-	75	-	-	-	80	-	-	-	85	-	-
	eGFR(CKD-EPI), mL/min/1.73m^2	88	-	-	102	-	93	-	-	-	84	-	91	-	-	-	88	-	-	-	82	-	-
250****591	sCr, µmol/L	65	74	56	36	43	43	69	52	97	-	81	117	132	132	145	-	170	180	100	96	145	-
	eGFR(CKD-EPI), mL/min/1.73m^2	103	98	110	132	123	123	101	113	75	-	93	60	52	52	46	-	38	36	72	76	45	-
250****542	sCr, µmol/L	89	-	-	93	-	124	137	-	117	78	84	79	86	-	-	82	-	-	-	-	80	-
	eGFR(CKD-EPI), mL/min/1.73m^2	74	-	-	70	-	49	44	-	53	85	79	85	77	-	-	81	-	-	-	-	84	-

Continued

Patient ID	Days after admission	22	23	24	25	26	27	28	29	30	31	32	33	34	35	36	37	38	39	40	41	……
250****341	sCr, µmol/L	-	80	110	-	-	-	-	-	-	-	-	-	-	-	-	-	-	-	-	-	-
	eGFR(CKD-EPI), mL/min/1.73m^2	-	62	42	-	-	-	-	-	-	-	-	-	-	-	-	-	-	-	-	-	-
250****870	sCr, µmol/L	-	42	47	-	-	-	-	-	-	-	-	-	-	-	-	-	-	-	-	-	-
	eGFR(CKD-EPI), mL/min/1.73m^2	-	98	94	-	-	-	-	-	-	-	-	-	-	-	-	-	-	-	-	-	-
250****193	sCr, µmol/L	-	-	76	-	90	-	105	-	-	121	82	63	65	-	104	131	-	105	-	98	-
	eGFR(CKD-EPI), mL/min/1.73m^2	-	-	90	-	76	-	63	-	-	53	86	98	96	-	64	49	-	63	-	69	-
250****591	sCr, µmol/L	-	-	-	-	-	-	-	-	-	-	-	-	-	-	-	-	-	-	-	-	-
	eGFR(CKD-EPI), mL/min/1.73m^2	-	-	-	-	-	-	-	-	-	-	-	-	-	-	-	-	-	-	-	-	-
250****542	sCr, µmol/L	-	-	77	-	-	-	-	-	-	-	-	-	-	-	92	-	-	-	-	-	-
	eGFR(CKD-EPI), mL/min/1.73m^2	-	-	86	-	-	-	-	-	-	-	-	-	-	-	71	-	-	-	-	-	-

sCr, serum creatine; eGFR, estimated glomerular filtration rate; CKD-EPI, Chronic Kidney Disease Epidemiology Collaboration.
